# Metformin attenuates sepsis-induced neuronal injury and cognitive impairment

**DOI:** 10.1186/s12868-021-00683-8

**Published:** 2021-12-15

**Authors:** Zhenghui Qin, Chenliang Zhou, Xiaochan Xiao, Cuiping Guo

**Affiliations:** 1 Department of Critical Care Medicine, Renmin Hospital of Wuhan University, Wuhan, China; 2 Department of Critical Care Medicine, Tianyou Hospital, Wuhan University of Science and Technology, Wuhan, China

**Keywords:** Sepsis, Cecum ligation and puncture, Metformin, Cognitive impairment, Inflammatory factors

## Abstract

**Background:**

Sepsis is considered to be a high-risk factor for cognitive impairment in the brain. The purpose of our study is to explore whether sepsis causes cognitive impairment and try to evaluate the underlying mechanisms and intervention measures.

**Methods:**

Here, we used cecum ligation and puncture (CLP) to simulate sepsis. Open field, Novel Objective Recognition, and Morris Water Maze Test were used to detect cognitive function, long-term potentiation was used to assess of synaptic plasticity, and molecular biological technics were used to assess synaptic proteins, ELISA kits were used to detect inflammatory factors. Metformin was injected into the lateral ventricle of SD rats, and we evaluated whether metformin alleviated CLP-mediated cognitive impairment using behavioral, electrophysiological and molecular biological technology experiments.

**Results:**

Here we report hippocampal-dependent cognitive deficits and synaptic dysfunction induced by the CLP, accompanied by a significant increase in inflammatory factors. At the same time, metformin was able to improve cognitive impairment induced by CLP in adult male rats.

**Conclusion:**

These findings highlight a novel pathogenic mechanism of sepsis-related cognitive impairment through activation of inflammatory factors, and these are blocked by metformin to attenuate sepsis-induced neuronal injury and cognitive impairment.

**Supplementary Information:**

The online version contains supplementary material available at 10.1186/s12868-021-00683-8.

## Introduction

Despite a rapid development in the sepsis treatment strategies during the past few years, the incidence and mortality of sepsis in clinical settings are still on the rise [1, 2]. Current drug development is mainly focused on regulating systemic inflammatory response, and coagulation and immune dysfunction, restoring pro-inflammatory and anti-inflammatory homeostasis, and improving patient prognosis [3, 4]. Even after anti-inflammatory therapy, patients with sepsis still face serious long-term problems such as physical, psychological, and cognitive impairment [5, 6]. Therefore, we speculated that sepsis is not only a process of systemic inflammatory response or immune disorder, but also involves functional changes in multiple organs in the body, including inflammatory response imbalance, immune dysregulation, mitochondrial damage, coagulation, autophagy, and neurological dysfunctions, endoplasmic reticulum stress, and ultimately lead to organ dysfunction. More and more clinical attention has been paid to the prognosis of cognitive function in patients with sepsis after extensive use of antibiotics [7–9]. Therefore, it is urgent to explore the pathogenesis of cognitive impairment and its molecular mechanism in sepsis patients and seek for feasible intervention methods.

Using the classical cecum ligation and puncture model to mimic sepsis [10–12], we found that cecum ligation and puncture led to learning and memory disorders, accompanied by the release of a large number of inflammatory factors. Metformin has anti-anxiety and anti-depression effects in patients with type 2 diabetes [13, 14]. A large body of literature has shown that the metformin was effective in regulating neurodegeneration because of its anti-apoptotic and antioxidant properties [15, 16]. Metformin could potentially combat certain neurotoxins and malign effects of certain neurodegenerative diseases [17, 18]. Therefore, we hypothesized that metformin has a certain therapeutic potential for cognitive impairment, and might alleviate sepsis-induced cognitive impairment.

In the current study, behavioral tests showed that metformin alleviated sepsis-induced learning and memory dysfunction in rats. In addition, metformin reduced inflammation in the hippocampus, restored synaptic protein expression, and improved synaptic damage. These results suggest that intervention with metformin may be helpful improving learning and memory function following sepsis induced neuronal injury and cognitive impairment, and provide a basis for clinical management of sepsis.

## Material and methods

### Reagents

Rat IL-1β (Interleukin 1 Beta) ELISA Kit (E-EL-R0012c), IL-6 (Interleukin 6) ELISA Kit (E-EL-M0044c), IL-17 (Interleukin 17) ELISA Kit (E-EL-R0566c), TNF-α (Tumor Necrosis Factor Alpha) ELISA Kit (E-EL-R2856c), IFN-γ (interferon-γ)ELISA Kits (E-EL-R0009c) were purchased from Elabscience Biotechnology.

### Animals

Male Sprague–Dawley (SD) rats (8 weeks old) were purchased from Liaoning Changsheng Biotechnology Co., Ltd. The rats were fed a normal rat diet and kept in a 12-h cycle of light and 12 h of darkness, and drank water freely. The rats were randomly divided into different experimental groups and treated according to the experimental requirements.

### CLP model

Rats were firstly anesthetized with 2–3% isoflurane. After anesthesia, the rats could breathe smoothly without turning over, and the depth of anesthesia was assessed via toe pinch. The skin was well cleaned and the area was draped. Sterile gloves were then worn when performing this procedure. A longitudinal incision of about 1 cm was made along the middle of the abdomen, sterile forceps was used to explore the abdomen and the cecum was pulled out. In sham operation group, only the cecum was pulled out of the abdominal cavity, and then the abdominal cavity was reduced. In CLP group, following the 1 cm incision cecum exposure, the cecum was then ligated with a 5/0 Prolene suture about 15 mm from the proximal cecum end, without clamping the cecum. The cecum was then perforated twice with 18G trocar, with taking care not to damage the mesentery and cecum blood vessels. After sending out little feces, the cecum was inserted back to the abdominal cavity and the abdominal wall incision was sutured layer by layer. All rats were given subcutaneous normal saline (10 ml) and analgesic buprenorphine (0.1 mg/kg) after operation. Rats were placed on hot pads until they recovered from anesthesia and returned to the feeding room for normal feeding. In our study, the mortality rate within 24 h with this CLP model is 10%.

Rats in each group were dynamically observed in 24 h after operation. In sham operation group, rats could move and eat normally after waking up, there was no significant change in urination and defecation, and the fatality rate was 0 in 24 h. In CLP group, the rats could move after waking up, but the rats showed drowsiness, crouching, hair standing up, urination and defecation were less than before operation, and the diet of food intake was reduced, the fatality rate was 10% in 24 h.

### Lateral ventricle stereotactic surgery

The rats were anesthetized with isoflurane and placed on stereotactic devices. The position of the stereodirectional drilling of the bilateral skull is as follows: 1.2 mm posterior, 2.6 mm lateral, 4.0 mm depth relative to bregma. Metformin (5 μL) was injected into the lateral ventricle at a rate of 0.25 μL/min. After injection, the needle was left in place to stay for 2 min and then slowly pulled out.

### Behavior tests

#### Open field test

The experimental test was carried out after the rats were bought to the laboratory and stayed for 2 day. The rats were placed in the laboratory chamber (100 × 100 × 70 cm container) for 5 min. The distance travelled and the number of times they crossed the intermediate area were tracked and measured to assess their motor ability.

#### Novel objective recognition test

The animals were placed in the laboratory and allowed to stay for 1 day, before the experimental test was carried out. Objects A and B were placed on two corners of the box, and the rat was placed in the center of the box, and allowed to explore freely for 5 minutes. Two hours later, object B was replaced by a new object C, and the rats were subsequently placed in the box for another 5 minutes. Twenty-four hours later, object C was replaced with object D and the rats were again placed in the box for 5 minutes. The exploration time of object A, B, C and D were recorded respectively.

#### Morris water maze test (MWM)

The MWM test was used to measure spatial learning and memory. The rats were put into the test room a day earlier. The rats found a hidden platform in a water maze within 60 s for 5 days, and if the rats did not find the platform within 60 s, they were guided to it and allowed to stay there for 20 s. On day 6, the platform was removed and spatial memory was tested by recording the times of crossing the platform position and the time spent by the rats in the target quadrant.

### Long-term potentiation (LTP)

LTP was used to detect synaptic plasticity in the hippocampus, which was detected by the MED64 multi-electrode array system (Alpha Med Sciences, Kadoma, Japan). The rats were quickly sacrificed and their brain tissue was quickly removed, and the brain slices were cut on a concussive sectioning machine. The slices were incubated in artificial cerebrospinal fluid (CSF) for 30 min, followed by three groups of high-frequency stimulation (HFS of 100 Hz, lasting for 1 s). The field excitatory post synaptic potentials (fEPSPs) were recorded.

### Western blotting

Tissue samples were lysed with RIPA containing the protease inhibitor PMSF and cocktail, on ice water and centrifuged at 12,000*g* for 10 min. The supernatant was collected, boiled, and the proteins were separated by SDS-PAGE and transferred to the nitrocellulose membrane. The membrane was blocked with 5% skimmed milk powder, the protein was labeled overnight with primary antibody at 4 °C. After 3 times wash with washing buffer, the second antibody was incubated at room temperature for 1 h followed by another 3 washes. The Odyssey system was used to observe the protein expression level. The primary antibodies used for Western blotting include: NR2A (1:1000; ab14596, Abcam, UK), NR2B (1:1000; SAB4300711; Millipore, USA), GluA1 (1:1000; 04-855, Millipore, USA), GluA2 (1:1000; MAB397, Millipore, USA), PSD95 (1:1000; 2507, Cell Signaling Technology, USA), Synapsin 1 (1:1000; AB1543, Millipore, USA), Synaptophysin (1:1000, s-5768,Sigma), Actin (1:1000; ab6276, Abcam), LaminB1 (1:1000; Abcam), NF-κB p65 (1:500; Cell Signaling Technology, USA).

The nuclear and cytoplasmic protein preparation kit (P1200, Pulilai) was used to separate the nuclear and cytoplasmic components according to the manufacturer’s procedures for subsequent experiments.

### Inflammatory factors were detected by ELISA

These kits use a double-antibody sandwich ELISA method to measure inflammatory factors in hippocampus tissues. The anti-rat IL-1β, IL-6, IL-17, TNF-α, IFN-γ antibodies were coated on an enzyme plate. During the experiment, the rat IL-1β, IL-6, IL-17, TNF-α, IFN-γ in the sample or standard product would bind to the coated antibody and the free components would be washed away. Biotinylated anti-rat IL-1β, IL-6, IL-17, TNF-α, IFN-γ antibodies and horseradish peroxidase labeled avidin were successively added. The anti-rat IL-1β, IL-6, IL-17, TNF-α, or IFN-γ antibody binds to rat’s IL-1β, IL-6, IL-17, TNF-α, IFN-γ bound to the coated antibody, and the biotin specifically binds to avidin to form an immune complex, and the free components are washed away. Color substrate (TMB) is added. This TMB becomes blue under the catalysis of horseradish peroxidase, and becomes yellow with the addition of termination solution. The concentration of IL-1β, IL-6, IL-17, TNF-α and IFN-γ in the sample are positively proportional to the OD450 value. The concentration of IL-1β, IL-6, IL-17, TNF-α, IFN-γ in the sample are calculated by drawing a standard curve.

### Statistical analysis

Data were expressed as mean  ±  SEM and analyzed using GraphPad statistical software. The one-way ANOVA was used to determine the differences among groups. For the comparison between two groups, the Student’s T test was used. The significance was assessed at *p*  < 0.05.

## Results

### Cecum ligation and puncture triggers cognitive deficits in rats

**Fig. 1:**
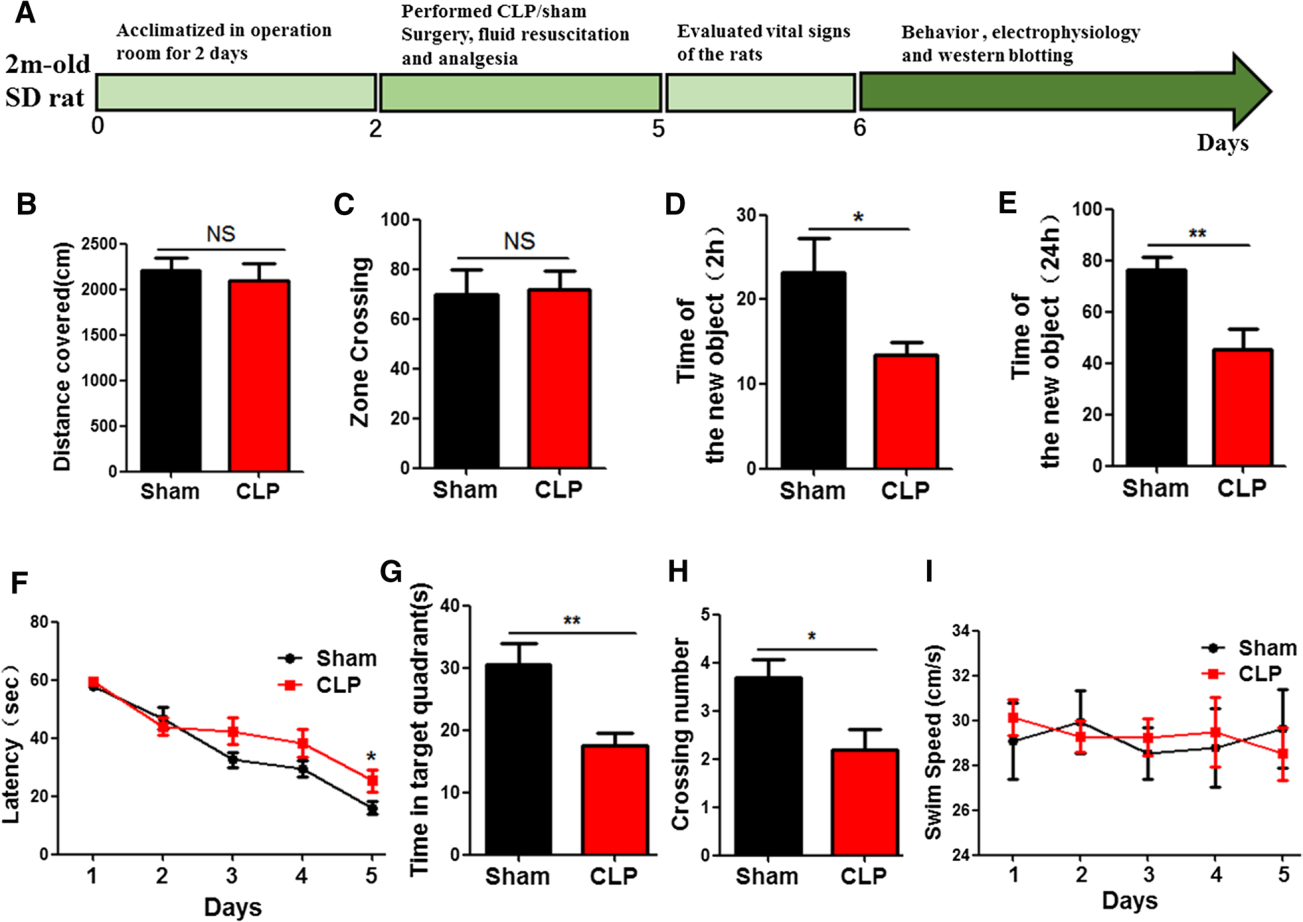
Cecum ligation and puncture triggers cognitive deficits in rats. **A** Experimental design sketch. The open field test results showed the total distance covered (**B**) and zone crossing (**C**). Novel object recognition test showed the time spent exploringthe new object at 2 hours (**D**) and 24 h (**E**). Morris water maze showed the latency to find the hidden platform (**F**). On day 6, with the platform removed, the time in the target quadrant (**G**), the number of target platform crossing (**H**) and swimming speed were analyzed (**I**). n  = 10. *p* value significance is calculated from T test, and data are represented as mean  ±  SEM. *p  < 0.05, **p  < 0.01 vs control group

To investigate the effect of CLP on cognitive function, 20 healthy SD rats were randomly divided into two groups. The CLP model group was established according to the classic model of sepsis, and the control group underwent sham operation as stated in the methodology. All rats were given subcutaneous normal saline (10 ml) and analgesic buprenorphine (0.1 mg/kg) after operation. Rats were placed on hot pads until they recovered from anesthesia and returned to the feeding room for normal feeding, followed by behavioral, electrophysiological, and biochemical tests (Fig. 1A). Behavioural tests were then carried out to assess cognitive function. The results from open field test showed that there was no significant difference in distance covered and zone crossing between the 2 groups (Fig. 1B, C). However, Novel Object Recognition (NOR) test showed that the exploration time of new objects in CLP group was significantly shortened at 2 h (Fig. 1D) and 24 h following the first exploration (Fig. 1E). Morris Water Maze (MWM) was used to test memory and learning function, and the result showed that the CLP group have a significantly increase in the latency of finding hidden platform (Fig. 1F). On day 6, the platform was removed for spatial memory test. Compared with the control group, both the time in the target quadrant (Fig. 1G) and the number of target platform crossings were reduced (Fig. 1H) in CLP group. But there was no significant difference in swimming speed (Fig. 1I). Taken together, these results suggest that CLP causes learning and memory impairments.

### Cecum ligation and puncture induces synaptic dysfunction in rats

**Fig. 2:**
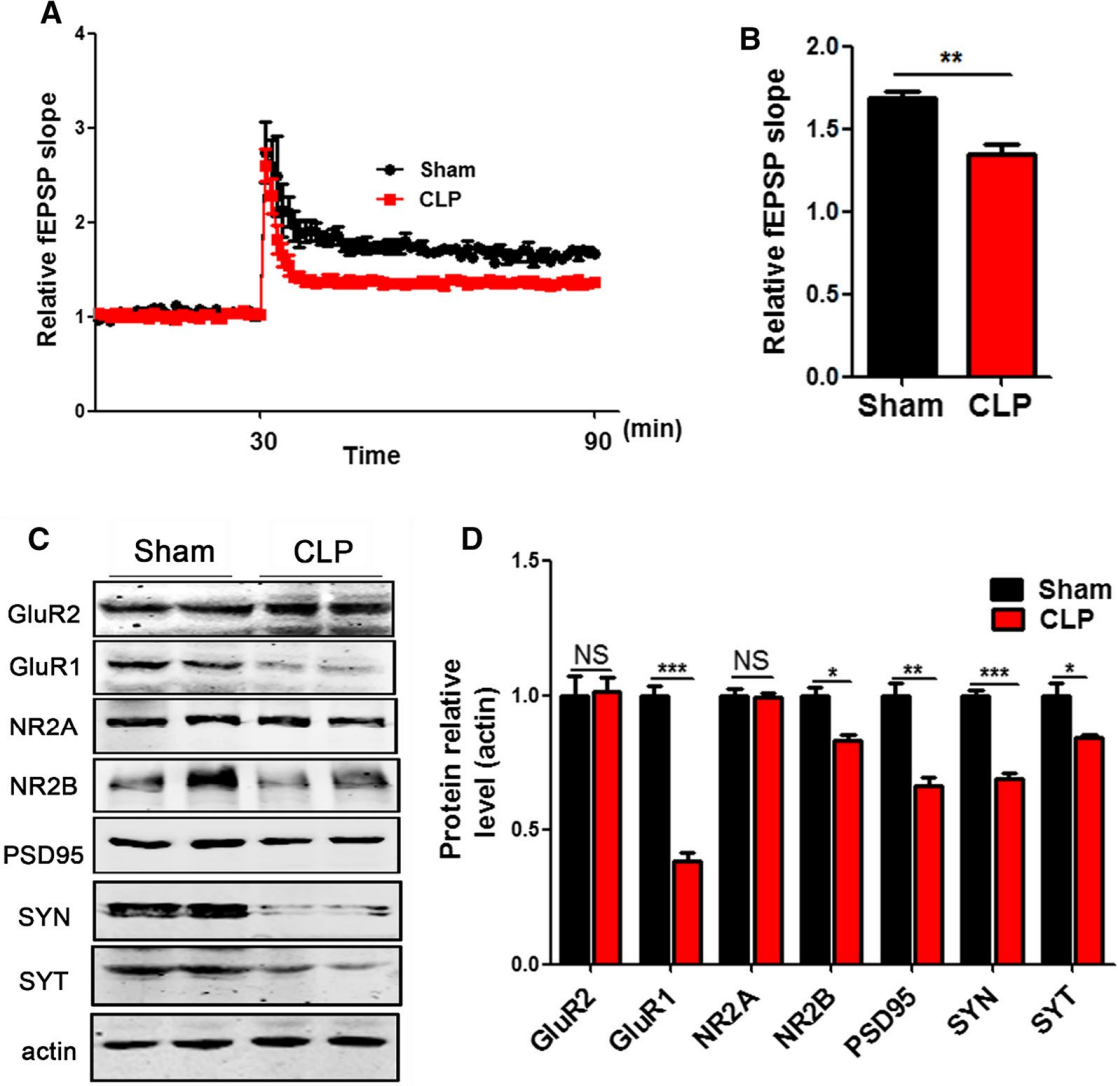
Cecum ligation and puncture induces synaptic dysfunction in rats. Hippocampal CA3-CA1 LTP and its quantification (**A**) were recorded using the MED64 system. Normalized CA3-CA1 fEPSP mean slope recorded from the CA1 dendritic region in hippocampal slices (**B**). **C**, **D** Brain tissues from hippocampi were homogenized, and synaptic protein levels were detected by immunoblotting. n  = 3. *p* value significance is calculated from T test, and data are represented as mean  ±  SEM. *p  < 0.05, **p  < 0.01 vs control group

It is known that the hippocampus (HIP) plays an important role in learning and memory function [19–22]. In order to investigate the underlying mechanisms how CLP induces learning and memory deficits, we explored the long-term potentiation (LTP) of the hippocampus which reflects synaptic plasticity [23, 24]. LTP test showed that the slope of the field excitatory postsynaptic potential (fEPSP) significantly decreased after high-frequency stimulation (HFS) in the CLP group (Fig. 2A, B), when compared with the control. In addition, Western blotting was used to test synaptic associated proteins in the hippocampus. The results (Fig. 2C) showed that presynaptic proteins Synapsin (SYN), Synaptophysin (SYT) and postsynaptic protein NR2B, GluR1, PSD95 were down-regulated in CLP group (Fig. 2D). Thus, we concluded that CLP leads to cognitive impairment probably via synaptic dysfunction which is reflected as a decrease in synaptic proteins.

### CLP upregulates the release of inflammatory factors

**Fig. 3:**
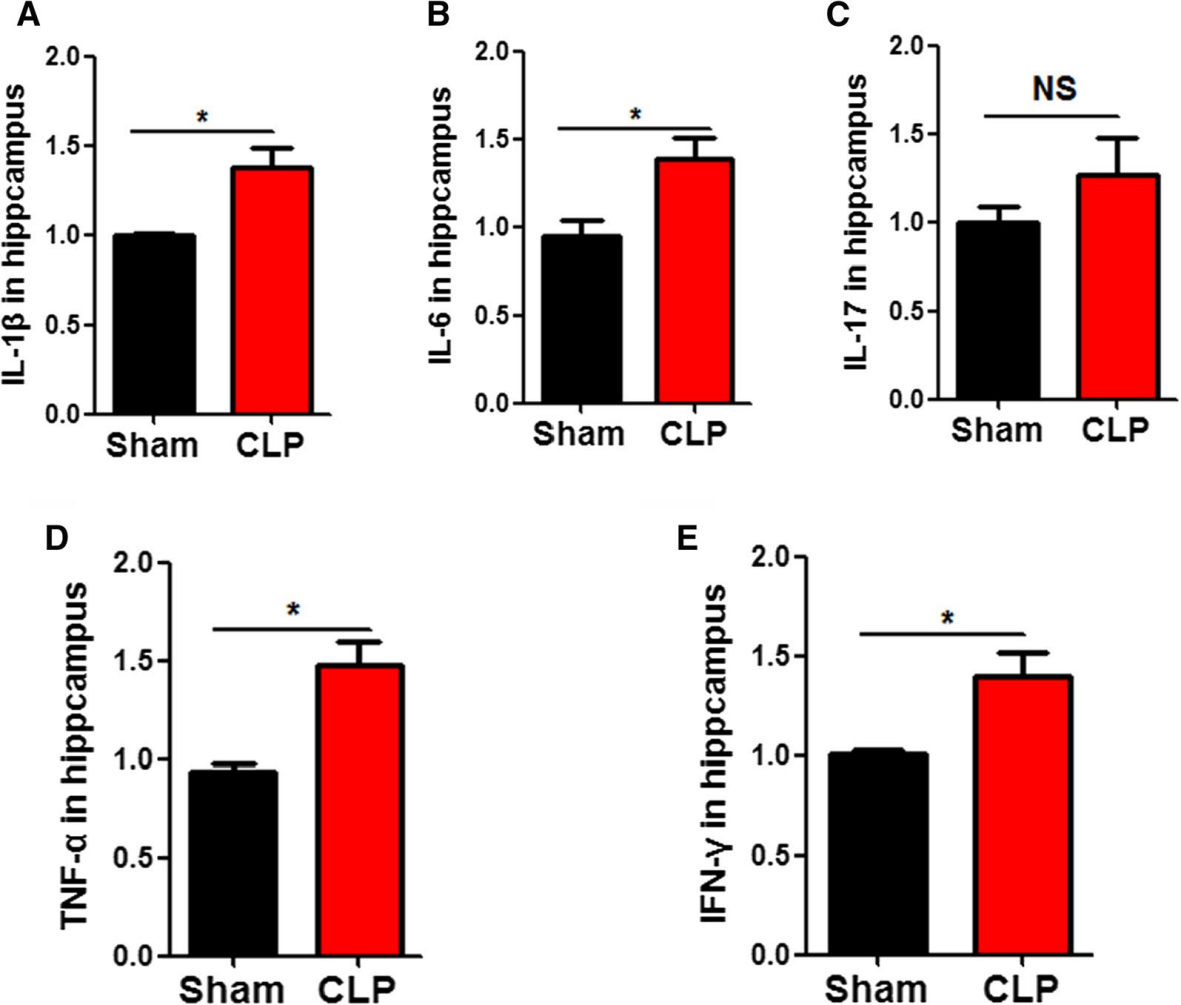
Cecum ligation and puncture upregulates the release of inflammatory factors. Enzyme-linked immunosorbent assay (ELISA) Kits were used to detect inflammatory factors including IL-1β (**A**), IL-6 (**B**), IL-17 (C),TNF-α (**D**), and IFN-γ (**E**). n  = 3. *p* value significance is calculated from a T test, and data are represented as mean  ±  SEM. *p  < 0.05, **p  < 0.01 vs control group

CLP causes a systemic inflammatory response that releases various immune factors and cytokines [11, 25]. To explore whether CLP could induce the release of these hippocampal immune factors, we used an enzyme-linked immunosorbent assay (ELISA) kit to detect these factors in hippocampal tissue lysates. The results showed that CLP led to a significantly increase in the inflammatory cytokines IL-1β (Fig. 3A), IL-6 (Fig. 3B), TNF-α (Fig. 3D), IFN-γ (Fig. 3E), but the level of IL-17 remains unchanged (Fig. 3C). These results suggest that CLP could activate or trigger the release of inflammatory factors in the hippocampus.

### Metformin alleviates CLP-induced cognitive impairment

**Fig. 4:**
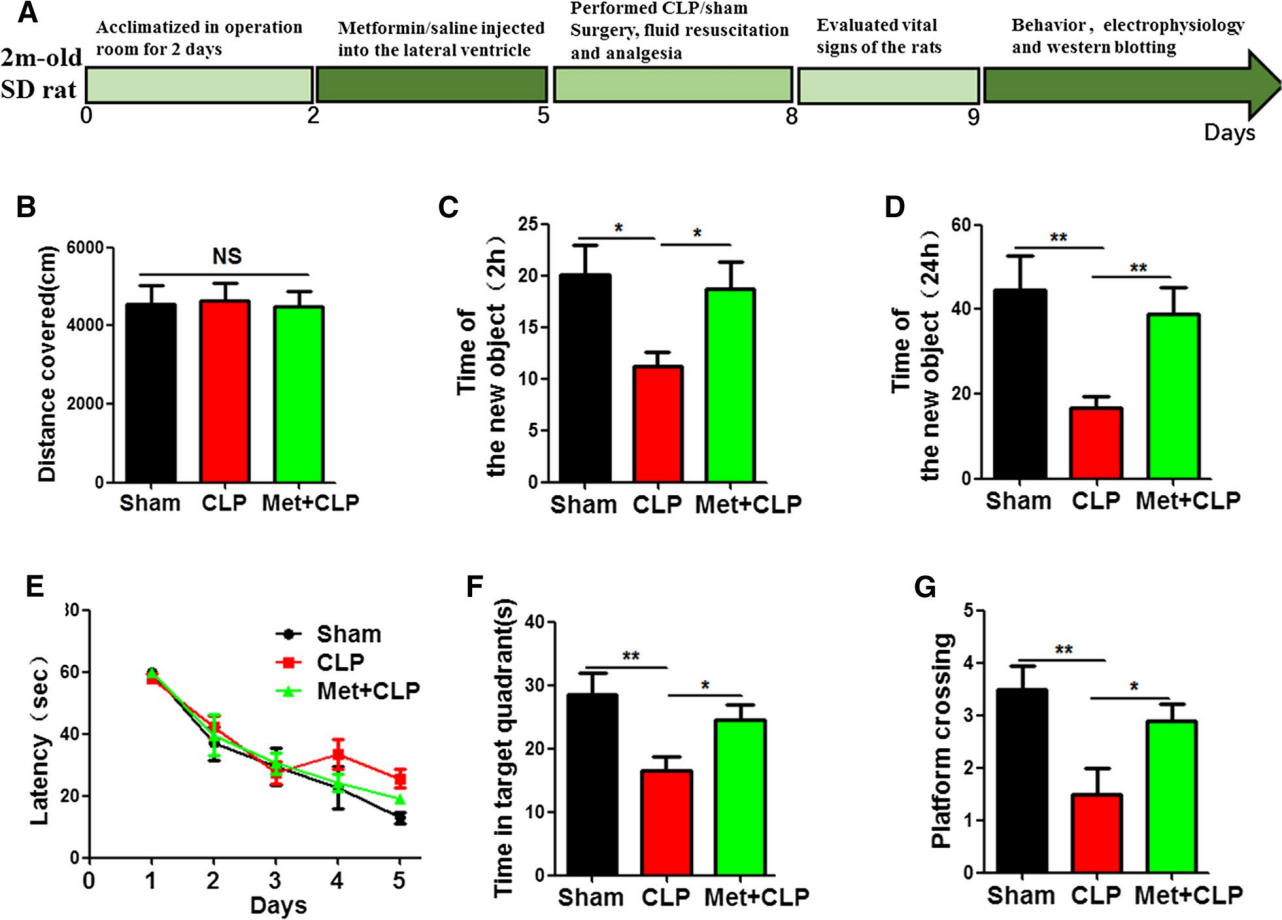
Metformin alleviates CLP-induced cognitive impairment. **A** Experimental design sketch. The open field results showed no significant difference in the total distance covered (**B**). Novel object recognition test (NOR) showed the measured recognition index of the new object at 2 h (**C**) and 24 h (**D**). Morris water maze (MWM) result showed the latency to find the hidden platform (**E**). On day 6, the platform was removed, and the time spent in the target quadrant (**F**), and the number of target platform crossing (**G**) were evaluated. n  = 10. *p* value significance is calculated from a one-way ANOVA or two-way ANOVA tests, all data represent mean  ±  SEM. *p  < 0.05, **p  < 0.01vs CLP group

Metformin has been reported to have anti-anxiety and anti-depression effects in patients with type 2 diabetes. A large body of literature has shown that metformin is effective in regulating neurodegeneration, therefore, we hypothesized that metformin might have a certain therapeutic potential for cognitive impairment. The result from our study revealed that metformin alleviated sepsis induced cognitive impairment. To further investigate on this, thirty healthy SD rats were randomly divided into 3 groups: the control group (sham group), model group (CLP group) and intervention group (Met  +  CLP group). The Met  +  CLP group were injected with metformin 10 mM into lateral ventricle. All rats were given subcutaneous normal saline (10 ml) and analgesic buprenorphine (0.1 mg/kg) after operation. Rats were placed on hot pads until they recovered from anesthesia and returned to the feeding room for normal feeding, followed by behavioral, electrophysiological, and biochemical tests (Fig. 4A). The results of open-field experiment showed that there was no significant difference covered in the total distance between the three groups (Fig. 4B). However, novelty recognition experiment showed that the Met  +  CLP group has a significantly increase in the time to explore the new object at both 2 h (Fig. 4C) and 24 h (Fig. 4D) when compared with the CLP group. Finally, Morris Water Maze (MWM) were was performed, and the results showed that the Met  +  CLP group has significantly reduced latency of finding the hidden platform (Fig. 4E). On day 6, we removed the platform for spatial memory test. Compared with the CLP group, the Met  +  CLP group spent more time in the target quadrant (Fig. 4F) and had increased number of target platform crossing (Fig. 4G). These data suggest that metformin is effective in restoring learning and memory impairment in CLP rat model.

### Metformin alleviates CLP-induced synaptic dysfunction

**Fig. 5:**
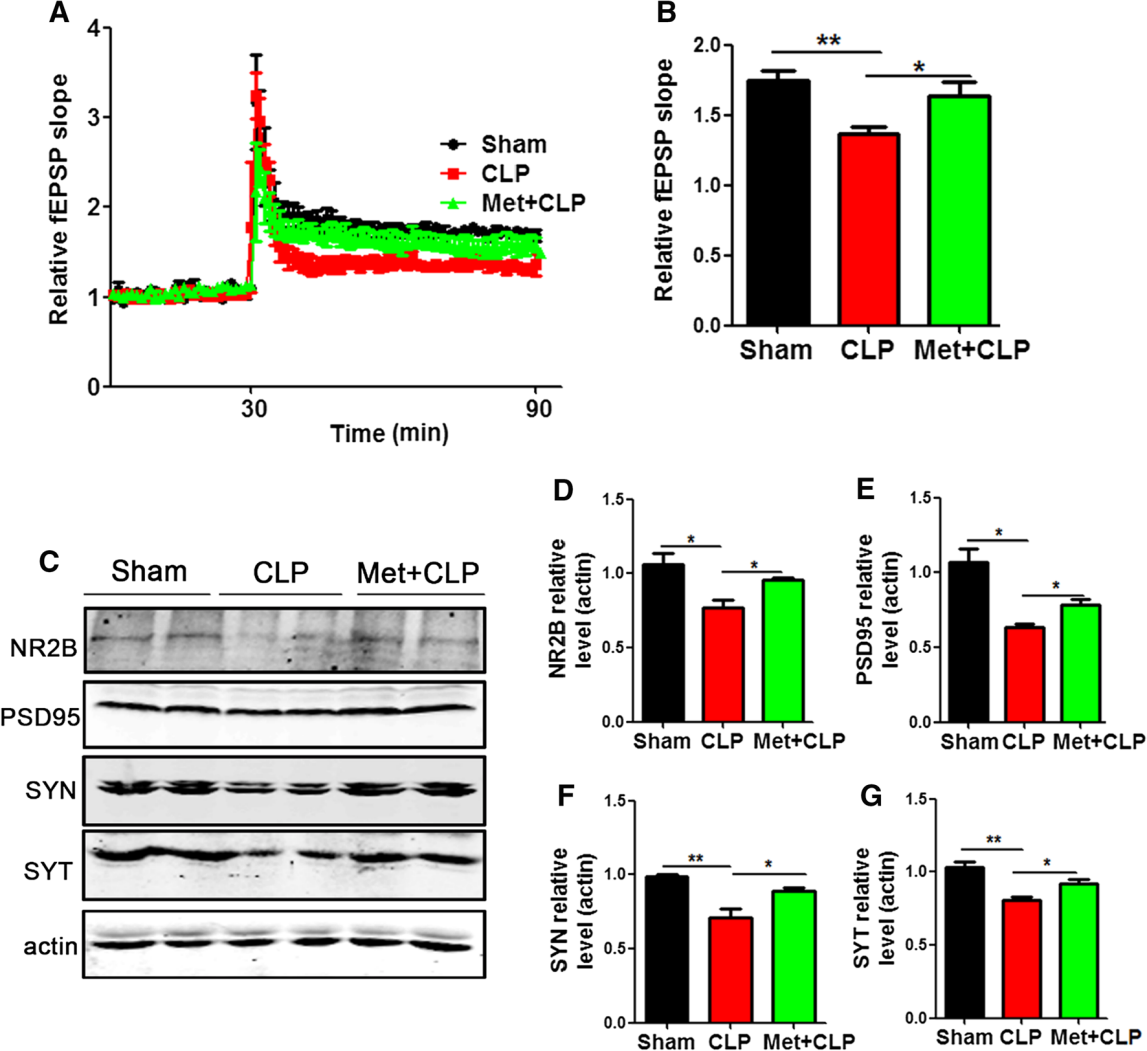
Metformin alleviates CLP-induced synaptic dysfunction. **A**, **B** CA3-CA1 fEPSP mean slope recorded from the CA1 dendritic region in hippocampal slices, n  = 3. Hippocampal tissues were homogenized, and synaptic protein levels were detected by immunoblotting (**C**). Postsynaptic proteins NR2B (**D**), PSD95 (**E**) and pre-synaptic proteins SYN (**F**), SYT (**G**) were evaluated. n  =  3. *p* value significance is calculated from a one-way ANOVA or two-way ANOVA tests, all data represent mean  ±  SEM. *p  < 0.05, **p  < 0.01 vs CLP group

We have shown that CLP synaptic plasticity impairments via dysregulation of synaptic proteins. We therefore wanted to see whether metformin could improve these alterations. LTP test showed that the slope of fEPSP in CLP group was lower than that in control group. However, this effect was significantly recovered in the Met  +  CLP group (Fig. 5A, B). Western blotting was used to detect synaptic proteins in the hippocampus (Fig. 5C), and it was found that the levels of NR2B (Fig. 5D), PSD95 (Fig. 5E), SYN (Fig. 5F), SYT (Fig. 5G) in the Met  +  CLP group were similar to those in the control group, while they were significantly reduced in the CLP group. These results suggest that metformin can abrogatesynaptic damage caused by CLP and improve cognitive function.

### Metformin pretreatment attenuates the release of inflammatory factors

**Fig. 6:**
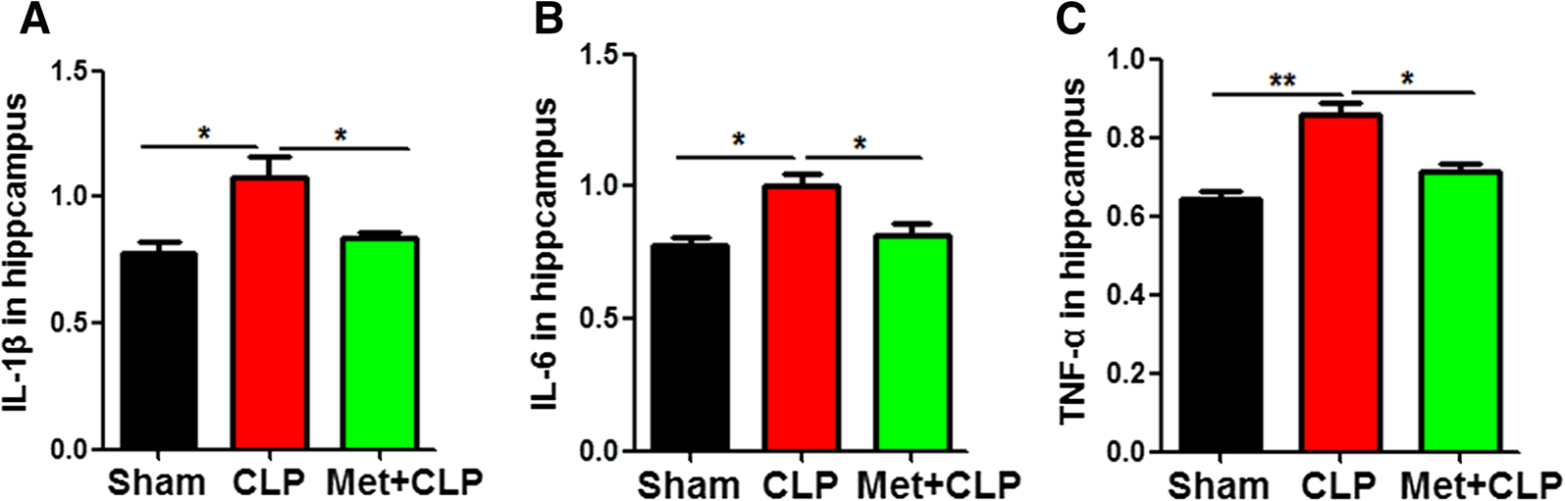
Metformin pretreatment attenuates the release of inflammatory factors. **A**–**C** Enzyme-linked immunosorbent assay (ELISA) Kits were used to detect inflammatory factors including IL-1β (**A**), IL-6 (**B**), and TNF-α (**C**). n  = 3. *p* value significance is calculated from a one-way ANOVA test, all data represent mean  ±  SEM. *p  < 0.05, **p  <  0.01 vs CLP group

It is suspected that CLP triggers the release of various pro-inflammatory mediators in the hippocampus to induce learning and memory impairments, while metformin restored cognitive impairment. In order to investigate the mechanism of metformin on learning and memory recovery in Met  +  CLP rats, we used enzyme-linked immunosorbent assay (ELISA) kit to detect inflammatory factors. The results showed that metformin significantly reduced the up-regulated inflammatory cytokines IL-1β (Fig. 6A), IL-6(Fig. 6B), and TNF-α (Fig. 6C) in the Met  +  CLP group compared with the CLP group. These data suggest that metformin intervention may alleviate CLP-induced neuron damage and cognitive impairment by reducing the release of inflammatory factors.

## Discussion

More and more data showed that the morbidity and mortality of sepsis in intensive care unit are increasing [26–28]. The use of antibiotics can effectively reduce the occurrence of systemic infection, but patients still face long-term and serious problems such as physical, psychological and cognitive impairments [6, 29]. We here used the classical CLP model to mimic sepsis, Novel Objective Recognition and Morris Water Maze experiment were employed to detect learning and memory impairments after CLP intervention which is accompanied by the release of a large number of inflammatory factors. Metformin has anti-anxiety and anti-depression effects in patients with type 2 diabetes [13]. Therefore, we hypothesized that metformin might have a certain therapeutic potential in cognitive impairment.

Thus, to verify our hypothesis, metformin was injected into the lateral ventricle, and the results showed that metformin significantly improved cognitive deficits and synaptic disorders in Met  +  CLP rats. It has been known that metformin plays an important role in lowering blood sugar and fighting inflammation, whereby it inhibits NF-κB signaling and reduces cellular inflammation [30–32]. Treatments that suppress the inflammatory process can be very beneficial in cognitive impairment setting as neuroinflammation was reported to play a key role in many neurodegenerative diseases [33, 34]. A key regulator of inflammatory processes is the nuclear factor κB (NF-κB) pathway. NF-κB regulates the expression of pro-inflammatory and pro-apoptotic genes in its active form. Molecules that interact with NF-κB, as well as subunits that make up NF-κB itself, represent therapeutic targets that can be modulated to reduce inflammation [35, 36]. The findings from our studies suggest that NF-κB may be an important metformin target for CLP-mediated cognitive impairment as metformin blocked the translocation of NF-κB from the cytoplasm to the nucleus.

Activation of NF-κB mediates the transfer of P65 from the cytoplasm to the nucleus, which in turn mediates immune responses and the release of pro-inflammatory mediators [37, 38]. Therefore, we further investigated whether CLP affects NF-κB expression. We isolated cytoplasmic and nuclear fractions and investigated the effect of metformin on nuclear NF-κB translocation by Western blotting. Compared with the CLP group, NF-κB protein level in cytoplasm was significantly increased in the Met  +  CLP group (Additional file 1: Fig. S1A, B), while nuclear NF-κB level was decreased in the Met  +  CLP group (Additional file 1: Fig. S1C, D). This suggests that metformin alleviates CLP-induced neuroinflammation by blocking the NF-κB cytoplasmic to nuclear translocation.

Our project is exploring molecular mechanisms. The molecular mechanism currently being explored is the activation of the NF-κB pathway after CLP surgery, which induces neuronal injury by activating the release of activated inflammatory factors. With the intervention of metformin, metformin inhibits the kinase JNK/AKT activity, thereby inhibiting the NF-κB pathway and improving cognitive impairment, so we need to improve it based on this part of the mechanic research.

## Conclusion

In the present study, a large amount of data confirmed that CLP could induce learning and memory dysfunction in rats. Metformin injection into the lateral ventricle could reduce inflammation in the hippocampus, restore synaptic protein expression, and improve synaptic damage. These results suggest that metformin therapy may be helpful for the recovery of learning and memory function after sepsis-induced neuronal injury and cognitive impairment, and provide a basis for clinical intervention in the complications of sepsis.

## Supplementary Information

**Additional file 1.** Metformin pretreatment attenuates the release of inflammatory factors via preventing the activation of the NF-κB pathway.

## Data Availability

The datasets used and/or analyzed during the present study are available from the corresponding author on reasonable request.

## Abbreviations

CLP: Cecum ligation and puncture
SD: Sprague–Dawley
Hip: Hippocampus
Met: Metformin
LTP: Long-term potentiation
SC: Schaffer collateral
fEPSP: Field excitatory postsynaptic potential
HFS: High-frequency stimulation
PSD95: Postsynaptic density protein 95
SYN: Synapsin
SYT: Synaptophysin
ELISA: Enzyme-linked immunosorbent assay
IL-1β: Interleukin-1β
IL-6: Interleukin-6
TNF-α: Tumor necrosis factor-α
IFN-γ: Interferon-γ
CA1: Cornu Ammonis area 1
CA3: Cornu Ammonis area 3
MWM: Morris water maze
NOR: Novel objective recognition
OF: Open field

## References

[1] Huang M, Cai S, Su J. The pathogenesis of sepsis and potential therapeutic targets. Int J Mol Sci 2019;20(21). 10.3390/ijms20215376 PMC6862039 31671729

[2] van der Poll T, van de Veerdonk FL, Scicluna BP, Netea MG (2017). The immunopathology of sepsis and potential therapeutic targets. Nat Rev Immunol.

[3] Salomao R, Ferreira BL, Salomao MC, Santos SS, Azevedo LCP, Brunialti MKC (2019). Sepsis: evolving concepts and challenges. Braz J Med Biol Res.

[4] Gotts JE, Matthay MA (2016). Sepsis: pathophysiology and clinical management. BMJ.

[5] Thompson K, Venkatesh B, Finfer S (2019). Sepsis and septic shock: current approaches to management. Intern Med J.

[6] Jones C, Griffiths RD (2013). Mental and physical disability after sepsis. Minerva Anestesiol.

[7] Bateman RM, Sharpe MD, Jagger JE, Ellis CG, Solé-Violán J, López-Rodríguez M, Herrera-Ramos E, Ruíz-Hernández J, Borderías L, Horcajada J, González-Quevedo N (2016). 36th International Symposium on Intensive Care and Emergency Medicine: Brussels, Belgium. Crit Care.

[8] Ni M, Meng X, Wang L, Zhao Y, Yu M, Shi S (2020). Polymyxin B-induced rhabdomyolysis: a case report. Medicine.

[9] Tauber SC, Djukic M, Gossner J, Eiffert H, Bruck W, Nau R (2021). Sepsis-associated encephalopathy and septic encephalitis: an update. Expert Rev Anti Infect Ther.

[10] Gong W, Wen H (2019). Sepsis induced by cecal ligation and puncture. Methods Mol Biol.

[11] Seemann S, Zohles F, Lupp A (2017). Comprehensive comparison of three different animal models for systemic inflammation. J Biomed Sci.

[12] Alverdy JC, Keskey R, Thewissen R. Can the cecal ligation and puncture model be repurposed to better inform therapy in human sepsis? Infect Immun. 2020;88(9). 10.1128/IAI.00942-19 PMC7440761 32571986

[13] Zemdegs J, Martin H, Pintana H, Bullich S, Manta S, Marques MA, Moro C, Laye S, Ducrocq F, Chattipakorn N (2019). Metformin promotes anxiolytic and antidepressant-like responses in insulin-resistant mice by decreasing circulating branched-chain amino acids. J Neurosci.

[14] McGettigan P, Roderick P, Mahajan R, Kadam A, Pollock AM (2015). Use of fixed dose combination (FDC) drugs in India: central regulatory approval and sales of FDCs containing non-steroidal anti-inflammatory drugs (NSAIDs), metformin, or psychotropic drugs. PLoS Med.

[15] Ahmadi A, Panahi Y, Johnston TP, Sahebkar A (2021). Antidiabetic drugs and oxidized low-density lipoprotein: a review of anti-atherosclerotic mechanisms. Pharmacol Res.

[16] Dludla PV, Muller CJF, Louw J, Mazibuko-Mbeje SE, Tiano L, Silvestri S, Orlando P, Marcheggiani F, Cirilli I, Chellan N et al. The combination effect of aspalathin and phenylpyruvic acid-2-O-beta-D-glucoside from rooibos against hyperglycemia-induced cardiac damage: an in vitro study. Nutrients 2020;12(4). 10.3390/nu12041151 PMC7231041 32325968

[17] El Massry M, Alaeddine LM, Ali L, Saad C, Eid AA (2021). Metformin: a growing journey from glycemic control to the treatment of Alzheimer’s disease and depression. Curr Med Chem.

[18] Rotermund C, Machetanz G, Fitzgerald JC (2018). The therapeutic potential of metformin in neurodegenerative diseases. Front Endocrinol.

[19] Goode TD, Tanaka KZ, Sahay A, McHugh TJ (2020). An integrated index: engrams, place cells, and hippocampal memory. Neuron.

[20] Jeneson A, Squire LR (2012). Working memory, long-term memory, and medial temporal lobe function. Learn Mem.

[21] Cope EC, Gould E (2019). Adult neurogenesis, glia, and the extracellular matrix. Cell Stem Cell.

[22] Goncalves JT, Schafer ST, Gage FH (2016). Adult neurogenesis in the hippocampus: from stem cells to behavior. Cell.

[23] Weston NM, Rolfe AT, Freelin AH, Reeves TM, Sun D (2021). Traumatic brain injury modifies synaptic plasticity in newly-generated granule cells of the adult hippocampus. Exp Neurol.

[24] Pigott BM, Garthwaite J (2016). Nitric oxide is required for L-type Ca(2+) channel-dependent long-term potentiation in the hippocampus. Front Synaptic Neurosci.

[25] Hwang JS, Kim KH, Park J, Kim SM, Cho H, Lee Y, Han IO (2019). Glucosamine improves survival in a mouse model of sepsis and attenuates sepsis-induced lung injury and inflammation. J Biol Chem.

[26] Zaragoza R, Ramirez P, Lopez-Pueyo MJ (2014). Nosocomial infections in intensive care units. Enferm Infecc Microbiol Clin.

[27] Schefold JC, Bierbrauer J, Weber-Carstens S (2010). Intensive care unit-acquired weakness (ICUAW) and muscle wasting in critically ill patients with severe sepsis and septic shock. J Cachexia Sarcopenia Muscle.

[28] Boeddha NP, Schlapbach LJ, Driessen GJ, Herberg JA, Rivero-Calle I, Cebey-Lopez M, Klobassa DS, Philipsen R, de Groot R, Inwald DP (2018). Mortality and morbidity in community-acquired sepsis in European pediatric intensive care units: a prospective cohort study from the European Childhood Life-threatening Infectious Disease Study (EUCLIDS). Crit Care.

[29] Konig C, Matt B, Kortgen A, Turnbull AE, Hartog CS (2019). What matters most to sepsis survivors: a qualitative analysis to identify specific health-related quality of life domains. Qual Life Res.

[30] Du RW, Bu WG (2020). Metformin improves depressive-like symptoms in mice via inhibition of peripheral and central NF-kappaB-NLRP3 inflammation activation. Exp Brain Res.

[31] Saisho Y (2015). Metformin and inflammation: its potential beyond glucose-lowering effect. Endocr Metab Immune Disord Drug Targets.

[32] He L (2020). Metformin and systemic metabolism. Trends Pharmacol Sci.

[33] Kwon HS, Koh SH (2020). Neuroinflammation in neurodegenerative disorders: the roles of microglia and astrocytes. Transl Neurodegener.

[34] Chen WW, Zhang X, Huang WJ (2016). Role of neuroinflammation in neurodegenerative diseases (review). Mol Med Rep.

[35] Kumar V (2019). Toll-like receptors in the pathogenesis of neuroinflammation. J Neuroimmunol.

[36] Shabab T, Khanabdali R, Moghadamtousi SZ, Kadir HA, Mohan G (2017). Neuroinflammation pathways: a general review. Int J Neurosci.

[37] Korbecki J, Bobinski R, Dutka M (2019). Self-regulation of the inflammatory response by peroxisome proliferator-activated receptors. Inflamm Res.

[38] Mendes KL, Lelis DF, Santos SHS (2017). Nuclear sirtuins and inflammatory signaling pathways. Cytokine Growth Factor Rev.

