# Supplementary material for: Metformin attenuates sepsis-induced neuronal injury and cognitive impairment
Source: BMC Neurosci. 2021 Dec 15;22:78. doi: 10.1186/s12868-021-00683-8 (PMC8675518; doi:10.1186/s12868-021-00683-8)
Supplement: Supplementary file 1 — Additional file 1. Metformin pretreatment attenuates the release of inflammatory factors via preventing the activation of the NF-κB pathway. [file 12868_2021_683_MOESM1_ESM.docx]

**Additional Figure 1**

**
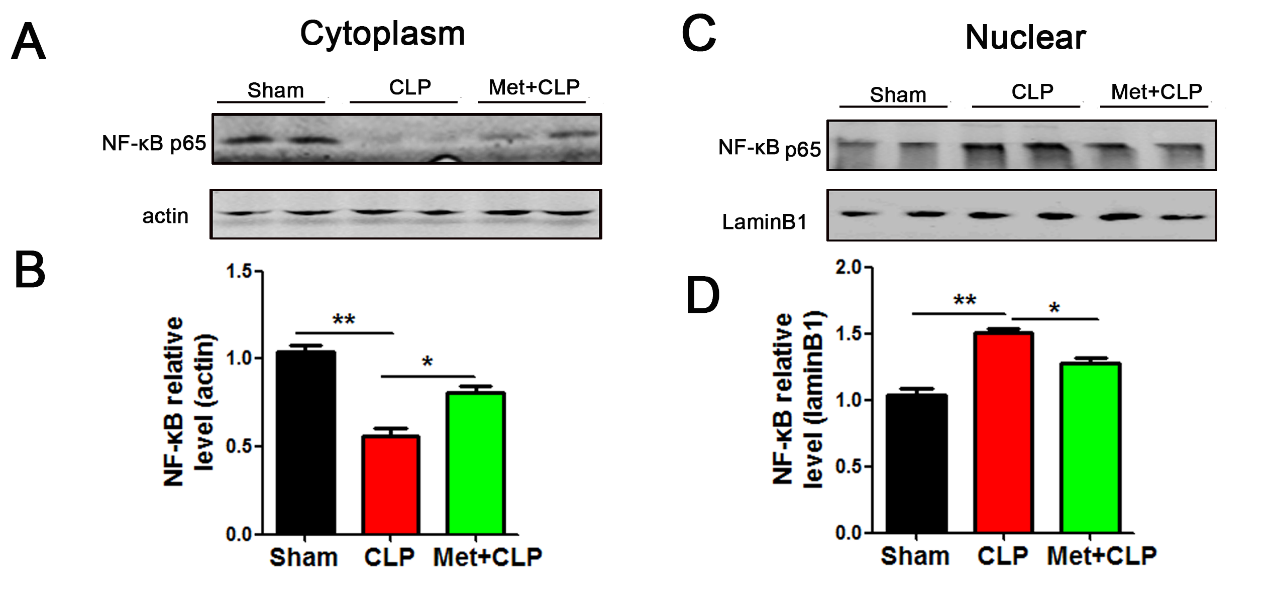
**

**Figure S1. Metformin pretreatment attenuates the release of inflammatory factors via preventing the activation of the NF-κB pathway.**

We separate cytosolic and nuclear proteins: NF-κB in cytoplasm (A, B) and in nucleus (C, D) were measured. n=3. *p* value significance is calculated from a one-way ANOVA test, all data represent mean ± SEM. **p* < 0.05, ***p* < 0.01 vs CLP group.
